# Chronic neutrophilic leukemia complicated with monoclonal gammopathy of undetermined significance: A case report and literature review

**DOI:** 10.1002/jcla.24287

**Published:** 2022-02-16

**Authors:** Jia‐Pei Gao, Li‐Jia Zhai, Xiao‐Hui Gao, Feng‐Ling Min

**Affiliations:** ^1^ Department of Hematology Affiliated Hospital of Yangzhou University Yangzhou University Yangzhou China

**Keywords:** chronic neutrophil leukemia, gene mutation, lymphocytic proliferative disease, next‐generation sequencing, plasma cell disorders

## Abstract

**Background:**

Study of the molecular biological characteristics of chronic neutrophilic leukemia complicated with plasma cell disorder (CNL‐PCD) and lymphocytic proliferative disease (CNL‐LPD).

**Methods:**

The clinical data of a patient with chronic neutrophilic leukemia complicated with monoclonal gammopathy of undetermined significance (CNL‐MGUS) in our hospital were reviewed, and the Chinese and/or English literature about CNL‐PCD and CNL‐LPD in PubMed and the Chinese database CNKI in the past 10 years was searched to analyze the molecular biological characteristics of this disease.

**Results:**

A 73‐year‐old male had persistent leukocytosis for 18 months. The white blood cell count was 46.77 × 109/L and primarily composed of mature neutrophils; hemoglobin: 77 g/L; platelet count: 189 × 109/L. Serum immunofixation electrophoresis showed IgG‐λ monoclonal M protein. A CT scan showed splenomegaly. Next‐generation sequencing (NGS) showed that CSF3R T618I, ASXL1 and RUNX1 mutations were positive. It was diagnosed as CNL‐MGUS. We summarized 10 cases of CNL‐PCD and 1 case of CNL‐LPD who underwent genetic mutation detection reported in the literature. The CSF3R mutational frequency (7/11, 63.6%) was lower than that of isolated CNL. The ASXL1 mutations were all positive (3/3), which may represent a poor prognostic factor. The SETBP1 mutation may promote the progression of CNL‐PCD. We also found JAK2, RUNX1, NRAS, etc. in CNL‐PCD.

**Conclusions:**

Chronic neutrophilic leukemia may be more inclined to coexist with plasma cell disorder. The CSF3R mutation in CNL‐PCD is still the most common mutated gene compared with isolated CNL. Mutations in SETBP1 and ASXL1 may be poor prognostic factors for CNL‐PCD.

## 1 INTRODUCTION

Chronic neutrophilic leukemia (CNL) is a rare *BCR*‐*ABL*‐negative myeloproliferative neoplasm (MPN). Some neoplasms, including plasma cell disorders (PCDs), can be accompanied by neutrophil proliferation, which is usually a leukemoid reaction. PCD complicated with real CNL is rare. Due to the limitations of detection methods in the past, a considerable number of CNLs or CNLs complicated with other diseases were misdiagnosed. With the application of next‐generation sequencing (NGS) technology, it was found that the majority of CNL patients harbored colony‐stimulating factor 3 receptor (*CSF3R*) mutations, and thus, the *CSF3R* mutation was regarded as an important diagnostic criterion of CNL.

Here, the clinical data of a patient with chronic neutrophilic leukemia complicated with monoclonal gammopathy of undetermined significance (CNL‐MGUS) in our hospital were analyzed. At the same time, 10 cases of CNL complicated with plasma cell disorder (CNL‐PCD) and 1 case of CNL complicated with lymphocytic proliferative disease (CNL‐LPD) with gene detection were searched from PubMed and the Chinese database CNKI.^1^, ^2^, ^3^, ^4^, ^5^, ^6^, ^7^, ^8^, ^9^, ^10^ We analyzed the molecular biological characteristics of these cases with the hope of further improving our understanding of this disease.

## 2 CASE PRESENTATION

A 73‐year‐old male patient was admitted to our hospital on November 9, 2019, due to persistent leukocytosis for 18 months and fatigue and night sweats for 1 month. The complete blood count (CBC) is described as follows: white blood cell count (WBC): 46.77 × 10^9^/L, neutrophils: 90.9%, lymphocytes: 3.1%, monocytes: 3.2%, eosinophils: 0.1%, basophils: 2.7%, red blood cell (RBC): 2.14 × 10^12^/L, hemoglobin(Hb): 77 g/L, and platelet count (PLT): 189 × 10^9^/L. A peripheral blood smear showed that neutrophils accounted for 84.1%, most of which were mature neutrophils. Serum immunofixation electrophoresis showed IgG‐λ type M protein, the M spike was 1.3 g/dL, serum κ free light chain 864 mg/dL, serum λ free light chain 2260 mg/dL, and κ/λ ratio of 0.38. The abdominal CT scan showed splenomegaly, skeletal CT survey including head, cervical, thoracic, and lumbar spine were done which showed no lytic lesions. Chromosome detection was as follows: 47, XY, and +21. Polymerase chain reaction results for the *BCR*‐*ABL* fusion, *JAK2*, *MPL*, and *CALR* genes were negative. NGS showed that *CSF3R T618I* (variant allele frequency [VAF], 48.7%) and *ASXL1* and *RUNX1* mutations were positive. After the bone marrow was sorted by CD138, fluorescence in situ hybridization (FISH) showed 15% positivity for 13q14 deletion, 13% positivity for Rb1 deletion, 15% positivity for IgH rearrangement (signal showed 1 yellow 1 green), 1q21 amplification, and P53 deletion negative. Flow cytometry analysis of 0.38% of clonal plasma cells in the bone marrow, and the results of immunophenotyping were as follows: cKappa‐/cLambda+/CD19‐/CD56‐/CD117‐/CD138+/CD27‐/CD38++/CD81+/CD45±. He was diagnosed with CNL‐MGUS. After admission, hydroxyurea was used to control the proliferation of leukocytes, febuxostat was used to reduce uric acid, and symptomatic and supportive treatment was administered. However, the patient had a progressive increase in leukocytes, the symptoms of night sweats and fatigue were not obviously improved, and progressive weight loss occurred. On June 13, 2020, he was hospitalized again because of abdominal pain. The abdominal CT showed a low‐density shadow of the spleen, suggesting spleen infarction. Repeat NGS showed *ASXL1 G635fs* (VAF 32.2%), *CSF3R T618I* (VAF 44.6%), and *NRAS G12A* (VAF 2.8%). He was treated with low‐molecular‐weight heparin anticoagulation for more than 1 month. After the symptoms of abdominal pain improved, the patient was treated with ruxolitinib at 15 mg twice daily, in addition to 1 g of hydroxyurea once daily on August 8, 2020. The patient's symptoms of night sweats and fatigue improved, leukocyte proliferation was significantly inhibited, the hemoglobin gradually increased, and no significant changes in bone marrow or immunoglobulin were found after re‐examination. A CT of the abdomen showed that the spleen had shrunk compared to before. He is now in stable condition and is under follow‐up.

## 3 DISCUSSION

Chronic neutrophilic leukemia is a rare *BCR*‐*ABL* negative MPN. In 2013, Maxson et al. found *CSF3R* mutations in 89% of CNL cases.^11^ Due to the limitations in understanding the disease in the past, some early literature reported that CNL‐PCD may be a leukemoid reaction caused by PCD, not real CNL.^12^ Myeloma cells and the abnormal immunoglobulins produced by them can stimulate bone marrow stromal cells to produce large amounts of cytokines (such as IL‐6) and stimulate the proliferation of granulocytes.^13^ Nagai detected the concentration of G‐CSF in the peripheral blood of a patient with PCD with “CNL.” The neutrophil count was proportional to the concentration of G‐CSF in serum.^14^ Therefore, patients with monoclonal gammopathy of undetermined significance (MGUS) or multiple myeloma (MM) need to find evidence of myeloid cloning before CNL is diagnosed. We reported a case of elderly CNL‐MGUS. Although there are a few reports of CNL with PCD or lymphocytic proliferative disease (LPD) in the literature, most of them are MM or MGUS. In addition to our case, we collected 11 cases of PCD or LPD complicated with CNL with evidence of myeloid cloning in PubMed and CNKI (Table 1). From Table 1, we observe no CNL cases complicated with LPD except follicular lymphoma (FL). This phenomenon suggests that CNL may be more inclined to coexist with MGUS or MM instead of coexisting by chance. The common genetic susceptibility loci in MPN and MM, and chronic inflammatory bone marrow microenvironment in MPN that in favor of the proliferation of monoclonal B cells, may be the pathogenesis of concurrence of these two different diseases.^15^, ^16^, ^17^

**TABLE 1:** Gene mutations and clinical characteristics of 12 patients with CNL with PCD or LPD

Case	Age (years)/Sex	Types of PCD or LPD	Gene mutations and detection methods	Disease progression and reference
1	81/M	FL	*JAK2 V617F* (+) *CSF3R*, *SETBP1* (‐) (PCR, Sanger sequencing)	‐;^1^
2	81/M	IgG‐κ type SMM	*JAK2 V617F* (+) *BCR*‐*ABL* (‐) (HRM, Sanger sequencing)	‐;^2^
3	57/F	IgG‐κ type MGUS	*CSF3R T618I*, *CSF3R S783fs* (+) *JAK2 V617F*, *BCR*‐*ABL*, *FGFR1*, *PDGFRA*, *PDGFRB* (‐) (FISH, Sanger sequencing)	‐;^3^
4	69/M	IgA‐κ type MGUS	*SETBP1 D868N* (+) *BCR*‐*ABL*, *CSF3R*, *JAK2* (‐) (PCR, Sanger sequencing)	Blast crisis^4^
5	70/M	MGUS	*SETBP1 G870S* (+) *BCR*‐*ABL*, *CSF3R*, *JAK2* (‐) (PCR, Sanger sequencing)	AML^4^
6	58/M	IgA‐κ type MM	*CSF3R P733T* (+) *JAK2 V617F*, *BCR*‐*ABL*, *CSF3R T618I*, *SETBP1* (‐) (PCR, Sanger sequencing)	Relapse after 6 years of standardized chemotherapy for multiple myeloma^5^;
7	53/M	MGUS	*CSF3R*, *SETBP1* (+) *BCR*‐*ABL* (‐) (NGS)	After 1 month of ruxolitinib treatment, a repeat NGS suggested clonal evolution by new mutations for *SRSF2* and *RUNX1* detected, along with the loss of the mutations for *CSF3R* and *SETBP1* ^6^;
8	87/M	IgA‐λ type MM	*CSF3R*, *ASXL1* (+) *BCR*‐*ABL*, *JAK2*, *MPL*, *CALR*, *SETBP1* (‐) (NSG)	Refused treatment and died 4 months later^7^;
9	77/M	IgD‐λ type MM	*CSF3R T618I* (+) *JAK2 V617F*, *BCR*‐*ABL*, *PDGFRA*, *PDGFRB*, *FGFR1* (‐) (PCR, FISH, Sanger Sequencing)	‐;^8^
10	73/M	IgG‐κ type MGUS	ASXL1 G976*, *NRAS G12A* (+) *JAK2*, *SETBP1*, *BCR*‐*ABL*, *PDGFRA*, *PDGFRB*, *FGFR1*, *PCM1*‐*JAK2* (‐) (NGS)	‐;^9^
11	72/M	MM	*CSF3R T618I*, *SF3B1* (+) (No detection method was provided)	‐;^10^
12	73/M	IgG‐λ type MGUS	*CSF3R T618I*, *ASXL1*, *RUNX1* (+) *BCR*‐*ABL*, *JAK2*, *SETBP1*, *MPL*, *CALR* (‐) (PCR, NGS)	After 9 months of hydroxyurea treatment, a repeat NGS suggested clonal evolution by a new mutation for *NRAS* detected and the loss of the mutation for *RUNX1*; Current report

Abbreviations: ‐, not given; CNL, chronic neutrophilic leukemia; FISH, fluorescence in situ hybridization; FL, follicular lymphoma; HRM, high‐resolution dissolution technology; LPD, lymphocytic proliferative disease; MGUS, monoclonal gammopathy of undetermined significance; MM, multiple myeloma; NGS, next‐generation sequencing; PCD, plasma cell disorders; PCR, polymerase chain reaction; SMM, smoldering multiple myeloma.

Among the 12 cases we summarized, 11 cases detected *CSF3R* mutation and 7 cases were positive. We found that *CSF3R* was the most common mutation among the 12 cases. However, the frequency of *CSF3R* mutations in CNL‐PCD (63.6% 7/11) was lower than that of isolated CNL reported in most of the literature.^18^, ^19^, ^20^, ^21^, ^22^, ^23^, ^24^ Whether the existence of both myeloid and lymphoid clones leads to a decrease in the mutation frequency of myeloid *CSF3R* mutation needs to be further clarified in more cases.

Table 1 shows that case 1 reported a case of CNL complicated with FL with the *JAK2 V617F* mutation in 2013, which was the first and currently only case that proves the coexistence of CNL and LPD.^1^ Case 2 reported a CNL patient complicated with smoldering multiple myeloma with the *JAK2 V617F* mutation in 2014. Although the author did not detect the *CSF3R* mutation, the *JAK2 V617F* mutation in peripheral blood supports the diagnosis of myeloid leukemia.^2^ The *JAK2 V617F* mutation is more frequent in MPN, such as polycythemia vera (PV) and primary thrombocytosis (ET), and CNL is rarely seen. *JAK2* is widely distributed in the cytoplasm of somatic cells and participates in the signal transduction pathway of hematopoiesis and the immune system. Yin reported 4 cases of CNL and found that two of them had only the *JAK2 V617F* mutation. Although the *CSF3R* mutation is the main carcinogenic mutation of CNL, a small number of CNL patients may be caused by the *JAK2 V617F* mutation.^25^ Gajendra et al. determined that the *JAK2 V617F* mutation had no unfavorable effect on the progression and prognosis of CNL.^26^ In addition, there is a report that a CNL patient with the *JAK2 V617F* mutation survived for 96 months.^27^ In the 12 cases we reviewed, the prognostic significance of the *JAK2 V617F* mutation in these patients was not known because the survival time of these two patients was not given.

In the 12 cases we summarized, the positive rate of *SETBP1* mutation was 37.5% (3/8), which was close to the median frequency of *SETBP1* mutation in isolated CNL.^1^, ^18^, ^19^, ^20^, ^21^, ^22^, ^28^, ^29^ CNLs that harbor both *CSR3R* and *SETBP1* mutations or harbor both *ASXL1* and *SETBP1* mutations have a low overall survival rate.^1^, ^18^ Lasho reported a case of CNL carrying both *CSF3R* and *SETBP1* mutations, and neither hydroxyurea nor ruxolitinib were effective. In vitro experiments showed that the patient's neoplasm cells were insensitive to the JAK inhibitor fedrotinib.^23^ However, a meta‐analysis that included 3 reports totaling 56 cases of CNL indicated that the *SETBP1* mutation had no unfavorable effect on the prognosis of CNL.^30^ As shown in Table 1, two CNL‐MGUS patients with *SETBP1* mutations reported in case 4 and case 5 all had blastic evolution during follow‐up treatment with hydroxyurea and died soon after.^4^ After 1 month of ruxolitinib treatment for case 7, repeat NGS suggested clonal evolution by new mutations for *SRSF2* and *RUNX1* detected, and two previously mutated genes (*CSF3R* and *SETBP1*) disappeared.^6^

In Table 1, seven cases had *CSF3R*, *JAK2*, and *SETBP1* mutations detected by Sanger sequencing. NGS has the advantages of high throughput, high sensitivity, quantification, and low cost and has been increasingly used clinically in recent years. Zhang et al. analyzed the genomic landscape of 158 patients with neutrophilic leukemias (including 39 cases of CNL) through whole‐exome and RNA sequencing and found that 69.6% of cases harbored ≥1 signaling pathway driver mutation, most of which were JAK/STAT and RAS signaling pathways. *EZH2*, *SETBP1*, *TET2*, *U2AF1*, and *SF3B1* were acquired by an early founder clone, while *ASXL1*, *SRSF2*, *CSF3R*, *CBL*, and *NRAS* were acquired in later subclones in some cases.^29^ By studying the genomics of CNL and atypical chronic myeloid leukemia (aCML), Maxson thought that clonal hematopoiesis is initially related to epigenetics or splicing gene mutations, but acquisition of a signaling mutation gives the clonal hematopoietic population a distinctive cell lineage phenotype.^31^ Two patients reported in cases 7 and 12 acquired new gene mutations during the follow‐up process,^6^ and the 2 patients reported in cases 4 and 5 had blastic evolution during the treatment process,^4^ indicating that CNL‐PCD will undergo clonal evolution or blastic evolution. However, whether CNL‐PCD is the same as isolated CNL with similarities in clonal evolution or blastic evolution needs to be expanded for further analysis.

Three cases had myeloid mutations detected by NGS, as shown in Table 1. In addition to *CSF3R* mutations, *ASXL1*, *RUNX1*, *NRAS*, and other mutations were also detected in these cases. Among these patients, the mutation of epigenetic modification *ASXL1* was positive (3/3), and case 8 died quickly due to refusal of treatment.^7^ Case 10 developed hemopericardium during the course of the disease.^9^ The patient we reported had splenic infarction and cachexia before ruxolitinib was added to the treatment. In multivariable analysis, mutated *ASXL1* was independently predictive of shortened survival in CNL. Elliott observed that CNL patients who evolved into chronic myelomonocytic leukemia (CMML) all carried *ASXL1* mutations but did not carry *SETBP1* mutations.^19^ Szuber et al. found that both univariate and multivariate analyses showed that the presence of *ASXL1* mutations was significantly related to a reduction in CNL overall survival and incorporated *ASXL1* mutations into a prognostic scoring system.^20^ From the above‐mentioned CNL literature on *ASXL1* mutations and three cases we summarized, we speculate that the significance of *ASXL1* mutations is similar in isolated CNL and CNL‐PCD.

In summary, CNL is a rare disease, and CNL complicated with other diseases is even rarer. What is the relationship between CNL and PCD? Are they close relatives, distant relatives, or two independent diseases? Modern molecular gene detection provides an effective means for accurate diagnosis. With the continuous emergence of future advanced detection technologies such as NGS, as well as the accumulation of research on these cases, more related questions, such as pathogenesis, disease transformation, and prognosis factors, will be revealed.

## Data Availability

The data used to support the findings of this study are included within the article. And the raw data used to support the findings of this study are available from the corresponding author upon request.
